# Body‐Integrated, Enzyme‐Triggered Degradable, Silk‐Based Mechanical Sensors for Customized Health/Fitness Monitoring and In Situ Treatment

**DOI:** 10.1002/advs.201903802

**Published:** 2020-05-11

**Authors:** Shan Zhang, Zhitao Zhou, Junjie Zhong, Zhifeng Shi, Ying Mao, Tiger H. Tao

**Affiliations:** ^1^ State Key Laboratory of Transducer Technology Shanghai Institute of Microsystem and Information Technology Chinese Academy of Sciences Shanghai 200050 China; ^2^ School of Graduate Study University of Chinese Academy of Sciences Beijing 100049 China; ^3^ Department of Neurosurgery Huashan Hospital of Fudan University Shanghai 200040 China; ^4^ Center of Materials Science and Optoelectronics Engineering University of Chinese Academy of Sciences Beijing 100049 China; ^5^ School of Physical Science and Technology ShanghaiTech University Shanghai 200031 China; ^6^ Institute of Brain‐Intelligence Technology Zhangjiang Laboratory Shanghai 200031 China; ^7^ Department of Brain‐computer Interface Shanghai Research Center for Brain Science and Brain‐Inspired Intelligence Shanghai 200031 China

**Keywords:** degradable sensors, health monitoring, in situ treatments, mechanical sensors, silk hydrogels

## Abstract

Mechanical signals such as pressure and strain reflect important psychological and physiological states of the human body. Body‐integrated sensors, including skin‐mounted and surgically implanted ones, allow personalized health monitoring for the general population as well as patients. However, the development of such measuring devices has been hindered by the strict requirements for human‐biocompatible materials and the need for high performance sensors; most existing devices or sensors do not meet all the desired specifications. Here, a set of flexible, stretchable, wearable, implantable, and degradable mechanical sensors is reported with excellent mechanical robustness and compliance, outstanding biocompatibility, remotely‐triggered degradation, and excellent sensing performance, using a conductive silk fibroin hydrogel (CSFH). They can detect multiple mechanical signals such as pressure, strain, and bending angles. Moreover, combined with a drug‐loaded silk‐based microneedle array, sensor‐equipped devices are shown to be effective for real‐time monitoring and in situ treatment of epilepsy in a rodent model. These sensors offer potential applications in custom health monitoring wearables, and in situ treatment of chronic clinical disorders.

## Graphical abstract

A set of controlled degradable mechanical sensors are developed using carbon nanotubes‐doped conductive silk hydrogels. The sensors can real‐time monitor external forces such as pressure, tension, and bending by sensing the forces‐induced resistance change of their carbon nanotube networks, and simultaneously control drug release through laser radiation‐triggered rapid degradation of the hydrogel‐based sensors for in situ chronic disorders treatment.

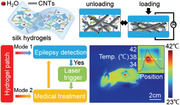

Body‐integrated devices and sensors are used, in real‐time, to monitor electronic,^[^ ^1^, ^2^ ^]^ mechanical,^[^ ^3^, ^4^, ^5^, ^6^, ^7^ ^]^ and biochemical^[^ ^8^ ^]^ signals of the human body. Additionally, a cluster of them (i.e., body‐NET),^[^ ^9^, ^10^, ^11^ ^]^ can be woven into clothes,^[^ ^3^ ^]^ worn on the skin,^[^ ^4^, ^5^, ^6^ ^]^ or implanted in the body^[^ ^7^, ^12^ ^]^ to monitor behavioral and physiological status intimately and continuously. Flexible, stretchable, and in some cases, degradable or self‐healable sensors are desired to be able to sense and respond to touch, pressure, temperature, humidity, and light, as well as to chemical and biological signals (beyond our existing sensing capabilities). Therefore, they would greatly benefit patients (especially those with chronic diseases) and significantly improve their quality of life.

In particular, flexible mechanical sensors, such as pressure and strain sensors, are predominantly important and have been intensively investigated among electronic skin and implantable devices; many psychological or physiological states of human body can be discerned from mechanical signals (e.g., blood pressure, pulse, and heart rate variability).^[^ ^3^, ^4^, ^6^, ^13^, ^14^, ^15^ ^]^ Though decent progress has been made in the development of mechanical sensors with sensitivity on par with human skin, further challenges remain with respect to improve the sensitivity, robustness, and to reduce adverse skin/body reaction when mounted or implanted for real‐world applications. For example, patients with epilepsy, a chronic non‐communicable brain disease that affects people of all ages, needs to be continuously monitored for signs and symptoms, and additionally, an immediate in situ treatment for seizures is critically important. However, most existing wearable devices and electronic skins can only fulfill the monitoring aspect,^[^ ^3^, ^4^, ^5^, ^6^ ^]^ thus limiting their applications. One of the key challenges lies in the materials requirement for body‐integrated devices; they need to be physically durable so that they can be worn on the skin or need to be well biocompatible so as to survive in the body after being implanted for months, if not longer. Ideally, materials of the substrate, encapsulation and passivation layers, and sensing components should all be functionalizable (electrically or biologically) so as to enable clinically or environmentally favorable therapeutic capabilities. For example, a method of controllable drug release and triggerable degradation is desired in order to reduce electronic waste and to eliminate the need for secondary surgery for retrieval of the implanted device.

In this context, silk proteins produced by silkworms and spiders, which are mechanically robust, biocompatible, and biodegradable,^[^ ^16^, ^17^ ^]^ fit well into this scope. Importantly, the mild condition for silk processing (into different material formats such as films, fibers, gels, and so on) enables to incorporate electrically or photonically functional, and optically or chemically active dopants (e.g., graphene, carbon nanotubes, laser dyes, metallic and semiconducting nanoparticles, and quantum dots) into the silk; especially, this allows to incorporate labile biological components (e.g., drugs, enzymes, antibodies, and antigens) into silk materials, which can reserve the bioactive functionality of components over extended timeframes.^[^ ^18^, ^19^, ^20^, ^21^ ^]^ Thus, this feature to impart additional components, in particular, provides a strategy to directly incorporate electrical features and biological activity into a bulk biopolymer‐based material substrate for multifunctional body‐integrated devices. For example, silk film, as a flexible, adhesive, and biodegradable substrate, can be patterned with silver nanowires electrodes and biochemical sensing modules to measure physiological signals.^[^ ^9^ ^]^ And silk solution could be directly doped with graphene to obtain conductive suspensions, which can be used to design electronic tattoos through screen printing or direct writing.^[^ ^22^ ^]^ Furthermore, silk nanofiber membranes can be carbonized to acquire electrical conductivity, thus enabling to serve as active materials for skin‐like pressure sensors.^[^ ^23^ ^]^

Herein, we report a set of flexible and stretchable sensors, using a conductive silk fibroin hydrogel (CSFH), for wearable and implantable applications. Benefiting from its superior flexibility and biocompatibility, the CSFH, doped with carbon nanotubes (CNTs) for desired conductivity, shows excellent flexibility with an elastic modulus of ≈0.001–0.15 MPa, high stretchability up to 100% strain, as well as good robustness and resilience. Because the CSFH exhibits characteristic responses to various simple and complex actions such as compression, tension, and bending alone or various combinations thereof, it can be used for sensing both pressure and strain and distinguishing different behavioral states. As a proof‐of‐concept, we demonstrated the capability of these devices for sign language translation and for monitoring physiological signals such as intracranial pressure (ICP)and muscle movement during speech or joint motions. Moreover, benefiting from the well‐known transient property of silk, the devices can be degraded on demand, through a light‐illumination‐triggered enzyme‐activated degradation/decomposition mechanism. Animal studies demonstrated the efficacy of our silk hydrogel sensor for epilepsy monitoring and in situ treatment, as combined with a drug‐loaded microneedle array, which was also made of silk proteins.

**Figure** **1**a presents a simple schematic diagram conceptually showing the basic structure and material components of CSFH. The secondary structure of silk protein consists of *α*‐helix, *β*‐sheet, random coil, and short polypeptide, all of which form the frame of silk hydrogel through covalent bonds and hydrogen bonds. Water molecules are stored among polyporous frames. CNTs are mixed into the hydrogel to produce electrical conductivity. The conductive CNT‐doped silk fibroin hydrogel is fabricated in a simple way by mixing and casting silk hydrogel precursors and CNT solution in a Petri dish (Figure S1, Supporting Information). The precursors, including hydrogen peroxide (H_2_O_2_), horseradish peroxidase (HRP), and silk solution, react to form stable and highly elastic hydrogels, through the well‐known enzyme catalyzed crosslinking of amino acid phenolic groups.^[^ ^24^, ^25^, ^26^ ^]^ 3D crosslinked networks formed by CNTs enhance the electrical conductivity and mechanical property of the CSFH. The CSFH exhibits excellent compressivity, stretchability, and bendability (Figure 1b), which are supported by the scanning electron microscope (SEM) images of cross sections of CSFHs under initial state, tension, compression, and bending states (Figure 1c). The SEM images in Figure 1c can also illustrate the sensing mechanism, which is due to the force‐dependent contact between CNTs when external forces are applied (Figure 1d). With tension applied, the internal gaps increase along the tensile direction (Figure 1c(ii)) compared to those at initial state (Figure 1c(i)), leading to less conductive pathways, thus the increase of resistance. In the unloading stage, the CSFH recovers to its initial state, causing an increase in the pathways between CNTs and thus resulting in a decrease in the resistance. In the same way, upon applying an external pressure, the internal gaps reduce considerably (Figure 1c(iii)) and more CNTs contact, resulting in more conductive pathways and a decrease in the resistance. Besides pressure and tension, the CSFHs are also sensitive to bending, thus enabling the possibility of wearable devices to detect joint motions. During joint bending (Figure 1c(iv)), the CSFH is subjected to a type of stretch, producing a tension force. An increase in the bending angle will produce a higher tension force, thus leading to an increase in the resistance. Figure 1e demonstrates a visual application of CSFH sensors, showing the fingers’ position‐based condition. A series of light‐emitting diode (LED) lights can be illuminated as programmed upon mechanical movements, with a visible fluctuation of their brightness upon bending or unbending of the CSFHs. Additionally, the CSFH is unique in its capability for controllable dissolution in the environment owing to the well‐known degradability of silk fibroin. Figure 1f shows a series of sequential images of CSFH upon insertion into a ≈0.25% papain (a temperature‐sensitive enzyme) solution to illustrate the progression of dissolving with respect to time.

**Figure 1:**
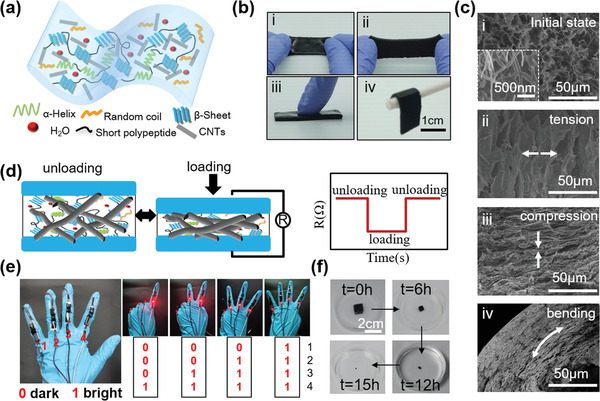
Degradable mechanical sensors using conductive silk fibroin hydrogels (CSFHs). a) Schematic diagram of the basic structure and the material components of the carbon nanotube (CNT)‐doped silk fibroin hydrogel. b) Photographs of the CSFHs under different deformations: i) initial state, ii) tension, iii) compression, and iv) bending, respectively. c) Representative SEM images of CSFHs under noted deformations. Inset in (i) show the 3D crosslinked networks of CNTs. d) Schematic illustration of the sensing mechanism: showing that resistance changes in response to the variation of the contact areas between CNTs, as caused by external forces. e) With the CSFH‐based mechanical sensors, the bending or unbending of fingers can be detected by the brightness of the LED. f) Dissolution of the CSFH in papain solution with concentration of 0.25%.

To assess the mechanical properties of the CSFH, we measured the compressive and tensile stress (the applied force divided by the film area) as a function of strain (the compressed/stretched distance divided by the film thickness/length). **Figure** **2**a presents a compressive stress plot of a typical CSFH sample, which has a thickness of ≈3 mm and an area of 1 cm^2^, against strain for five loading cycles. Those curves overlap with minimal hysteresis and no significant differences are observed, even for 100 cycles (Figure S2, Supporting Information). Hysteresis is defined as the maximum difference between the loading and unloading curve divided by the full‐scale output. The hysteresis of CSFH is found to be ≈10% at a pressure of 6 kPa, which is considerably lower than the hysteresis observed for many previously reported elastic pressure sensors.^[^ ^14^, ^27^ ^]^ It should be noted that hysteresis, in the mechanical response of CSFH to compression, is caused by viscoelasticity of the material and occurs in almost all compressible carbons.^[^ ^14^, ^28^, ^29^ ^]^ The compressive modulus, quantified as the tangent of the compressive stress–strain curve, is quite low (≈0.015 MPa) at low strain as desired and increases with compression (up to ≈0.15 MPa), while the tension modulus calculated from the curve of Figure 2b ranges from 0.001 to 0.01 MPa. Figure 2b exhibits the outstanding stretchability of the CSFH with a consistent rupture strain of 100%. Since both compression and tension moduli are lower than many elastic modulus values of degradable pressure and strain sensors reported previously, the CSFH satisfies the softness requirements for mechanical compliance.^[^ ^7^ ^]^

**Figure 2:**
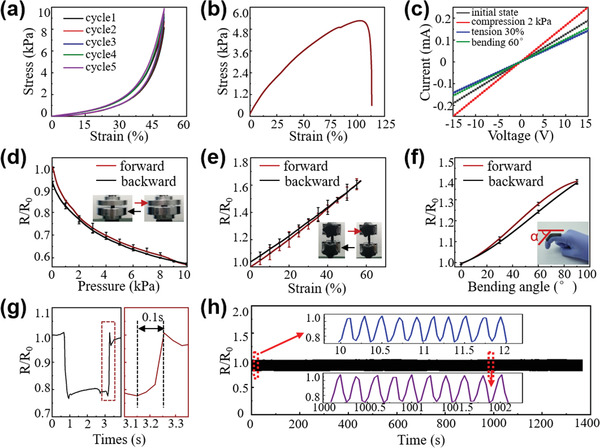
Mechanical properties and sensing performance of CSFH. a) Compression tests on a CSFH film with a thickness of ≈3 mm and an area of 1 cm^2^. b) The extension test on the CSFH film with a thickness of ≈600 µm, a length of ≈3 cm, and a width of ≈5 mm. The break point shows up at 100% strain. c) *I*–*V* curves of the CSFH under different deformations. Electrical resistance response to d) pressure, e) tension strain, and f) bending angles. g) Instant response of the CSFH‐based mechanical sensor, which exhibits a response time of ≈0.1 s. h) Long‐term durability test (1400 s, 5 Hz) of the CSFH‐based mechanical sensor at a pressure of 2 kPa. Inset: magnified view of ten cycles for the early and end stages, respectively.

In addition to mechanical properties, the current–voltage (*I*–*V*) characteristics of CSFH under different deformations were studied (Figure 2c; Figure S3, Supporting Information). Currents increased linearly with voltages no matter if the sample is under no deformation or in tension (30%), compression (2 kPa), or bending states (60°). A smooth, linear, characteristic *I*–*V* curve represents the excellent ideal Ohmic behavior of the CSFH, which is very desirable for its applications as pressure and strain sensors. The slopes of the *I*–*V* curves exhibit discrete characteristics under different deformations, indicating the resistances are distinctive under corresponding deformations; that is, the resistance is inversely proportional to the slope according to the Ohm's law. The difference in resistances between CSFH samples in Figure 2c and Figure S3, Supporting Information, is due to the difference in sizes of CSFH samples. It is clearly shown that the slope under an initial state is larger than slopes under tension and bending states but smaller than that under a compression state. Therefore, CSFHs under the initial state have a smaller resistance than those under the tension and bending states but larger than those under the compression state. Those results are consistent with the schematic described in Figure 1d.

Sensor performance under pressure, tension strain, and bending angles is illustrated in Figure 2d–f. Figure 2d depicts the piezoresistive response of the CSFH‐based pressure sensor, showing that the resistance ratio *R*/*R*_0_ (*R*_0_ is the initial electrical resistance and *R* is the changed electrical resistance at present) decreases with increasing pressure. The pressure sensitivity *S* can be defined as the slope of the resistance ratio versus pressure in Figure 2d (*S* = Δ(*R*/*R*_0_)/Δ*P*). The pressure sensor exhibits a high sensitivity to pressure <500 Pa, with *S* = ≈0.3 kPa^−1^, but the pressure sensitivity drops to 0.03–0.07 kPa^−1^ in the high‐pressure regime (3 kPa < *P* < 10 kPa), which is common in degradable pressure sensors.^[^ ^7^, ^30^, ^31^, ^32^ ^]^ This relationship between *S* and *P* is desirable in real‐world applications, because the progressive reduction of *S* offers high sensitivities to very low loads and a large detection range for higher pressure loads (for which high sensitivity is not required).^[^ ^4^, ^5^, ^27^, ^33^ ^]^ Figure 2e presents the strain sensor response curves of the CSFH, showing that the values of *R*/*R*_0_ increase almost linearly with the increasing tension strain. The average gauge factor (GF = Δ(*R*/*R*_0_)/Δ*Ɛ*, where *Ɛ* denotes the applied tension strain) of the CSFH‐based strain sensor is ≈1.1, which is higher than the gauge factor of degradable silicon‐based strain gauges reported previously.^[^ ^34^ ^]^ To evaluate the ability of CSFH to function as a bending‐sensitive sensor, we measured the values of resistance ratio *R*/*R*_0_ in response to various bending angles, with the device installed on the index finger joint to form the bending angle *α*. As shown in Figure 2f, the values of *R*/*R*_0_ continuously increase as bending angle increases with a satisfactory sensitivity of 0.0045 per degree, which is comparable to the most sensitive bendable sensor reported previously.^[^ ^14^ ^]^

To further investigate the properties of the CSFH‐based mechanical sensor, for demonstration purposes, we examined the instant response and cycling stability of the CSFH functioning as a pressure sensor. As illustrated in Figure 2g, the immediate response time of the pressure sensor is in a millisecond range. Figure 2h demonstrates the high durability, repeatability, and stability of the CSFH‐based pressure sensor under a pressure of 2 kPa, applied at a frequency of 5 Hz for >1400 s. The amplitude of *R*/*R*_0_ exhibits negligible changes after >7000 loading–unloading cycles. The fast response time and cycling durability meet the requirements for real‐time monitoring of physiological signals by wearable or implantable devices.

Benefiting from the well‐known transient property of silk,^[^ ^16^, ^17^ ^]^ the CSFH can also be degraded in response to a specific environmental condition. However, unlike the random coil to *β*‐sheet crosslinked silk film, which dissolves readily in deionized (DI) water at controlled rates, the enzymatically crosslinked CSFH is degradable through the assistance of a protease such as papain. Papain, purified from the ripening fruit of *Carica papaya*, is a pure natural enzyme and a kind of biocompatible protein, which has been widely used in medical field. For example, papain has been used for wounded tissues repair for many decades without any cytotoxicity and cutaneous irritation.^[^ ^35^, ^36^ ^]^ **Figure** **3**a depicts a schematic diagram of the controlled degradation of papain‐doped CSFH by an external trigger. When the CSFH is mixed with papain with low‐specificity, those bonds maintaining the structure of the CSFH and secondary structures of silk are destroyed. Eventually the frame is destroyed and the CSFH gradually degraded. For the papain‐undoped CSFH (Figure 1f), when it is immersed in papain solution, the degradation occurs from the surface and gradually diffuses to interior of the device. If the CSFH is doped with papain, degradation occurs throughout the whole device and exists all the time at an extreme low rate due to the intrinsic humid condition of CSFH, and the degradation rate increases when external triggers are applied. For example, once the papain‐doped CSFH is immersed into DI water, degradation rate would increase, caused by the replacement of the water molecules. In addition, the degradation of papain‐doped CSFH can be controlled by external temperature, due to the temperature‐sensitivity of papain. The internal polyporous structure of the papain‐doped CSFH is well‐organized initially and then becomes more and more disordered during the degradation process (Figure 3b). In order to evaluate the mechanical properties of the papain‐doped CSFH during the degradation, we measured the compressive and tensile stress–strain curves. We compared the compressive modulus (red column) and tensile modulus (blue column), defined as the slope for the first 10% strain (as calculated by a linear fit of the initial linear elastic region), at several stages of degradation as shown in Figure 3c. The compressive modulus and tensile modulus clearly decrease with the increase in degradation time, suggesting that the papain‐doped CSFH would deform more easily. The difference in compression and tension modulus resulted mainly from the difference in water content of the CSFH used in compression and tension test. In tension modulus test, the CSFH samples were immersed into DI water for 10 min to get higher tensile elongation, while the CSFH samples were directly measured for compression modulus. Figure S4, Supporting Information, explores the compression and tension modulus of the CSFH with the similar water content. Sensing performance during the degradation was also studied. We determined the resistance in response to a constant strain (20%), pressure (500 Pa), and bending angle (10°), respectively at the different stages of degradation. Figure 3d presents the absolute values of the change in ratio Δ*R*/*R*_0_, which do not show any significant decrease with the increase in degradation time; rather, they slightly increase until the papain‐doped CSFH is completely broken up, owing to that the part degradation of hydrogel frame and the disorder of the internal polyporous structure lead to decrease of the elastic modulus of the CSFH, thus the CSFH is easier to deform and more sensitive to mechanical forces. This result is desirable because it means the CSFH sensors maintain their sensing sensitivities during the whole degradation until completely broken up, which is comparable with other degradable sensors published previously.^[^ ^7^, ^12^ ^]^ The degradation rate of papain‐doped CSFH is also dependent on environmental conditions such as temperature and pH values, due to the enzymatic properties of papain. To investigate the effect of temperature and pH values on the degradation rate, nine papain‐doped CSFHs with the same size (10 mm long,10 mm wide, and 3 mm thick) were immersed in PBS solutions with a gradient of three temperatures in one direction and a gradient of pH values in the orthogonal direction. Figure 3e exhibits photographs of nine CSFHs before and after degradation for 1 h. The figure clearly shows that the size difference among residual CSFHs along a row is bigger than those along a column, indicating that temperature has a stronger effect on the degradation rate of CSFH than pH, corresponding with enzymatic activity of papain. It should be noted that the residual CSFH in the top right corner is clearly the smallest, suggesting the most suitable condition for degradation is at 50 °C, pH = 6. To exploit this property of temperature‐sensitive degradation, laser heating could be used to accelerate the local degradation rate of the CSFH. To further enhance the laser heating effect, gold nanoparticles (AuNPs) are doped into the CSFH to enable laser plasmon resonance, which induces more heating.^[^ ^21^, ^37^ ^]^ The effect of AuNPs on the local temperature of the CSFH is shown in Figure S5, Supporting Information, indicating that AuNPs make a clear impact. Figure 3f presents a schematic for a green laser (with wavelength *λ* = 532 nm)‐mediated heating of the AuNP‐doped CSFH network. In this experiment, the CSFH sample was illuminated simultaneously by two sets of green lasers with incident radiation powers of 100 and 50 mW (Figure S6, Supporting Information). The temperature distribution of the sample was acquired by an infrared camera after 5 min illumination and a stable equilibrium temperature was reached (Figure 3g). The absorption peaks at the specific positions of illumination show 24 and 13 °C measured temperature rises, respectively, as compared to other positions without illumination. The right panel of Figure 3g displays the photograph of a CSFH sample after 1 h degradation, clearly demonstrating that the illuminated spots are degraded faster than the rest of the hydrogel. Moreover, the difference in the mechanical properties between the CSFH samples with and without laser illumination further corroborates this laser‐triggered degradation. As shown in Figure S7, Supporting Information, the compressive modulus of sample 1 (illuminated by a laser for 5 min) is smaller than that of sample 2 (without illumination), consistent with the previous result.

**Figure 3:**
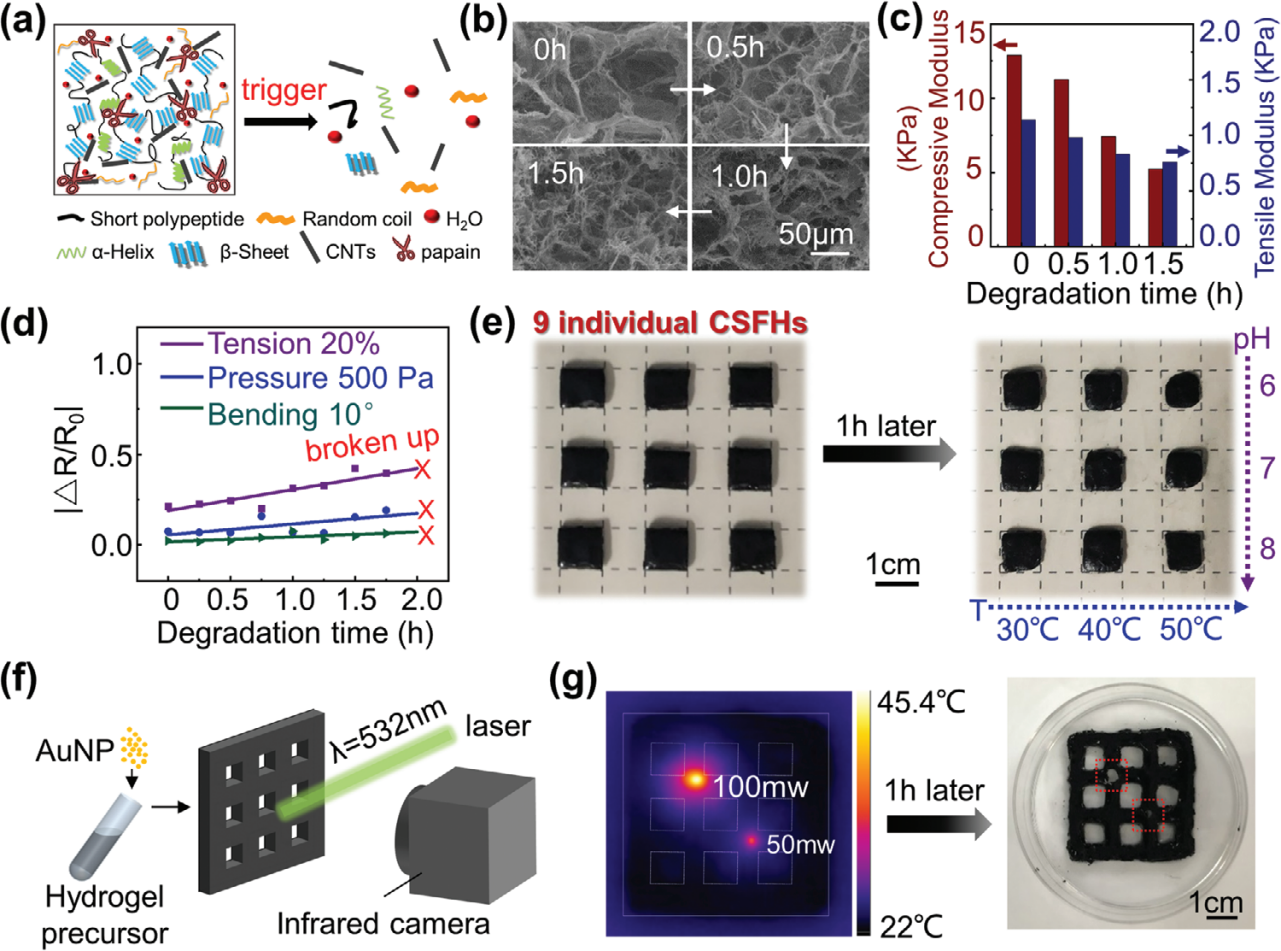
Degradation/decomposition characteristics of CSFH. a) Schematic diagram showing the controlled degradation mechanism, through adding papain into the CSFH. b) Representative SEM images collected at several stages of degradation for CSFH doped with papain. c) Compressive and tensile modulus of CSFH at different degradation times. d) Resistance response for constant pressure (500 Pa), tension (20%), and bending angle (10°) during degradation. e) Degradation/decomposition rate study at different temperature and pH. The result shows that the degradation rate reaches a maximum at a temperature of 50 °C and a pH of 6. f) Schematic illustration of visible‐light‐triggered degradation of AuNP‐doped CSFH. g) Thermal image of the CSFH treated by two 532 nm lasers with average radiation power of 100 and 50 mW for 5 min. After 1 h of radiation, the irradiated area is completely degraded.

Based on the excellent sensing performance and suitable mechanical properties of the CSFHs, a series of experiments were then conducted to explore their potential applications for sign language translation and real‐time monitoring of physiological signals. As shown in **Figure** **4**a, a total of 14 CSFH‐based mechanical sensors were installed on all the finger joints of a typical human hand; the intention was for complete sensing coverage of finger gestures during sign language communication. Each sensor, corresponding to the respective finger, are abbreviated as follows: thumb (T1, T2), index (I1, I2, I3), middle (M1, M2, M3), ring (R1, R2, R3), and pinky (P1, P2, P3) finger. The CSFH‐based mechanical sensors generate specific signals for joint bending while signing, based on the letters, words, and gestures in the conversation. As a demonstration, Figure 4a displays an experimental result for the word “hydrogel” using distinctive color mapping for each sensor and signal pattern of the gesture. In addition to sign language translation, the CSFH‐based mechanical sensor could also detect physiological signals such as ICP and muscle movement during speech or joint motions. As shown in Figure 4b, the CSFH film was assembled into a pressure sensor by sandwiching the film between two flexible, aluminum (Al)‐coated, conductive silk film electrodes and then was implanted in the intracranial space of Sprague‐Dawley rats to detect the ICP. In order to close the intracranial cavity, bone cement was used to seal the craniectomy defect, and conventional sutures were used to hold the surgical site closed in a standard process. The upper panel presents the experimental results of ICP monitoring during the normal state and after euthanasia. The change of resistance value (Δ*R*) shifts from a wave‐like pattern, rhythmically up and down, during normal state, to a straight line after euthanasia; this indicates the rhythmical fluctuation of ICP is associated with the cardiac impulse and breathing during the normal state. Thus, the signal is absent and ICP is flat after euthanasia. ICP is a vital indicator for physical condition because many cerebral diseases are known to lead to abnormal fluctuations of ICP.^[^ ^12^, ^38^, ^39^ ^]^ As a demonstration, ICP during epilepsy was monitored using the CSFH‐based pressure sensor in the bottom panel of Figure 4b. The value of Δ*R* decreases gradually once the epilepsy is induced by injecting penicillin G sodium (PNG) solution, suggesting that ICP increases during the epilepsy. The CSFH‐based mechanical sensor could also be attached onto a typical human throat to noninvasively monitor the muscle movement during speech, as shown in Figure 4c. The sensor exhibits high sensitivity and distinct peaks and valleys when the speaker vocalizes different words and phrases such as “ah,” “hello,” and “silk hydrogel”, respectively as labeled. Speech recognition via the relative positions of peaks and valleys is a common method for piezoresistive mechanical sensors.^[^ ^4^, ^40^ ^]^ Finally, for another direct human application, sporting motions were also monitored with a sandwich‐structured CSFH‐based mechanical sensor attached to a test subject's knee joint by tightly wrapping adhesive tape around the electrodes in order to avoid any slipping of the mounted sensor (Figure 4d). The knee, the largest joint in the human body, is essential for normal human locomotion, typically with some degree of bending or straightness. When the wearer lifts their foot off the ground, their knee bends, causing the sensor to stretch and its electrical resistance to increase. On the contrary, when the wearer returns to a standing posture, their knee straightens, allowing the sensor to recover and its relative resistance to return back to the initial value. As shown in Figure 4d, the sandwich‐structured sensor distinguishably responds to walking, jogging, and squatting with distinct amplitude and frequency values, owing to the different angles and bending frequency of the knee joint for each motion. The *R*/*R_0_* peak value is ≈1.6 for squatting, larger than those for both walking and jogging. However, the fluctuation frequency of *R*/*R_0_* for jogging is largest, indicating that the bending angles for squatting are the largest and that the knee‐bending for jogging is the fastest among the three motions. These experimental results on sensor performance meet the expectations. Overall, these experiments demonstrate the capability of CSFH‐based mechanical sensors for wearable individual‐centered health monitoring and for great potential to assist people with speech disorders to communicate with the world.

**Figure 4:**
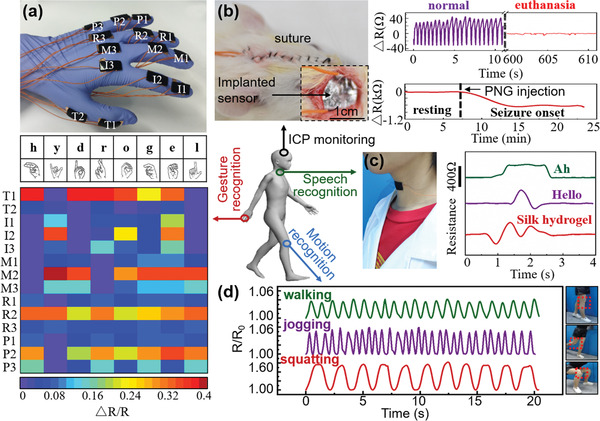
E‐health monitoring using CSFH‐based mechanical sensors. a) Gesture recognition using 14 CSFH‐based mechanical sensors on every joint of a human hand. Color mapping of the variation in output resistance change, from the 14 CSFH‐based mechanical sensors, can be used for translation of the word “hydrogel”. b) Intracranial pressure (ICP) monitoring by placement of the CSFH‐based pressure sensor in the intracranial space. Upper panel: ICP at normal state and after euthanasia. Bottom panel: Real‐time monitoring of the ICP during an induced epileptic seizure. c) Speech recognition of the words of “ah,” “hello,” and “silk hydrogel”. d) Motion recognition of walking, jogging, and squatting.

In addition to real‐time monitoring of physiological signals and sporting motions, the CSFH‐based mechanical sensor can also be loaded with therapeutic molecules to integrate a drug release function, benefiting from the fact that silk is a particularly promising biomatrix platform for stabilizing a range of labile biocompounds, even at elevated temperatures.^[^ ^1^, ^18^, ^19^, ^20^, ^21^ ^]^ The temperature‐sensitive degradation of CSFH is extra useful for controlling drug release in smart medical treatments, since the release rate could be controlled by environmental temperature as well as direct laser heating (Figure S8, Supporting Information). Epilepsy is one of the most common neurological disorders and can cause severe body convulsions. Thus, it was chosen for a proof‐of‐concept study here. Epilepsy treatment was used to verify the property of controllable drug release in order to explore CSFH's potential for in vivo applications. **Figure** **5**a illustrates the schematic of a laser heating‐triggered therapy using a patch formed by a CSFH‐based mechanical sensor in combination with microneedle arrays. Herein, the microneedle arrays, adhered tightly to the CSFH‐based mechanical sensor, carry a drug of interest, and assist its controlled release from the sensor into the body. The CNTs outside cannot penetrate the microneedles, because the silk microneedles can serve as a filter to separate the particles by their size (see details in Figure S9, Supporting Information).^[^ ^41^ ^]^ The patch here was loaded with phenobarbital, and then attached on the back of BALB/c Nude mice for health monitoring (Figure 5b). The patch is capable to detect seizures, as judged by the recorded motions and corresponding signal pattern caused by the symptoms that are differentiable from the healthy state. When the epileptic symptoms occur, the patch degradation can be triggered by laser heating, thus releasing the phenobarbital into the body for treatment (Figure 5c). Figure 5e presents the detected signals (Δ*R)* during the experiment. For the resting section, Δ*R* fluctuates slightly over time indicating the mouse breathes gently. After 20 min of monitoring, a penicillin solution was injected to properly induce the epilepsy seizure onset. Astonishing Δ*R* spikes occur, with up to fivefold increase in amplitude compared with the resting state, indicating the exquisite distinguishability of this biomedical sensor. Then laser heating was applied onto the patch by illuminating it with an incident radiation power of 100 mW. As shown in Figure 5d, the local temperature of patch reaches 42 °C. After 10 min of this light‐triggered medical treatment, the epileptic convulsion distinctively alleviates (Figure 5e). Note that the absorption peak of the AuNPs or gold nano‐shells can be adjusted for example, to match an infrared light source with better skin/organ penetration depth, if needed.

**Figure 5:**
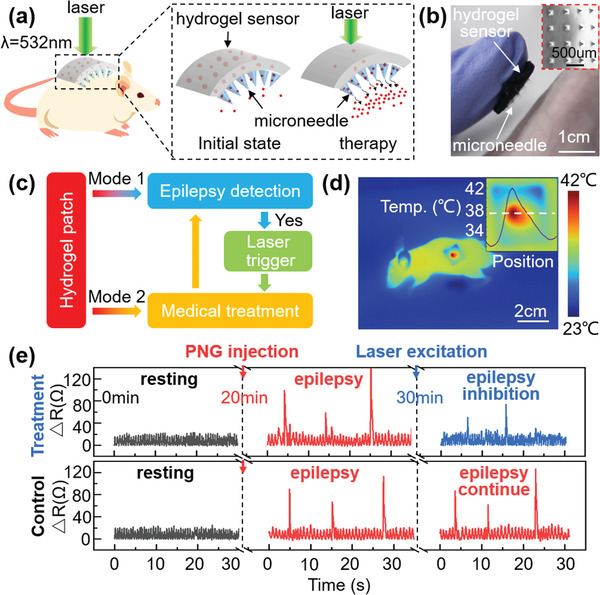
CSFH integrating with drug‐loaded silk microneedles to serve as a visible‐light‐triggered drug‐release patch. a) Diagram showing the visible‐light‐triggered drug‐release mechanism of the CSFH plus microneedles patch. b) Photograph of the patch located onto the backside of nude rat. Inset view shows a representative SEM image of the microneedles. c) Flow diagram for visible‐light‐triggered therapy via the patch. d) Thermal image of the patch on the backside of a nude rat after 5 min laser heating. Inset showing a magnified image of the patch and the corresponding temperature profile. e) Epilepsy detection and treatment. After the injection with penicillin G sodium solution for ≈20 min, anesthetized rats start to show intense convulsion symptoms, as represented by sudden large peaks in resistance change. Once the epilepsy symptom is detected, the patch is illuminated by a 532 nm laser with an average radiation power of 100 mW to release drugs for medical treatment. After 10 min of treatment, the rat in the experimental group exhibits effective epilepsy inhibition while the control group continues to have seizures.

In summary, we have demonstrated a set of flexible, stretchable, wearable, and implantable CSFH‐based mechanical sensors that can detect various mechanical signals such as pressure, strain, and bending angles. These silk‐based mechanical sensors have good mechanical compliance with respect to human skin and internal organs due to their small elastic modulus (0.001–0.15 Mpa) and good adhesiveness, robustness, and resilience properties. The sensitivity of bending angle monitoring is comparable to previously reported devices. Our devices can be stretched to 100% of their original length without significantly decreasing their sensing performance or undergoing permanent deformation. For proof‐of‐concept, it was shown that the devices can be applied onto many locations of the body for successful motion recognition (e.g., walking, jogging, squatting, fine finger gestures, and muscle movement during speech) and physiological signal monitoring (e.g., ICP and vocal cord muscle vibration). Furthermore, by integrating the thermo‐activated papain with the gold nanoparticle‐doped CSFH, the degradation/decomposition of CSFH can be triggered by light. Combined with a drug‐loaded microneedle array (which was also made of silk), the device shows high efficacy for real‐time monitoring and in situ treatment of epilepsy in a rodent model. These flexible mechanical sensors, based on our unique conductive silk fibroin hydrogel design, provide a close‐loop (i.e., “sense‐and‐respond”) multifunctional flexible electronics platform, offering numerous potential applications in soft robotics, clinical treatment, and patient rehabilitation.

## Experimental Section

##### Silk Solution Preparation

Bombyx mori silk fibroin was prepared with an established protocol. Bombyx mori cocoons were boiled in 0.02 m Na_2_CO_3_ (Sigma‐Aldrich, USA) aqueous solution for 30 min, and then rinsed with distilled water for three times (30 min per time) to remove the Na_2_CO_3_ and sericin. After dried in air for ≈12 h, the degummed cocoons were dissolved in 9.3 m LiBr (Sigma‐Aldrich, USA) solution at 60 °C for ≈4 h. This solution was dialyzed for 2 days in distilled water by using Slide‐A‐Lyzer dialysis cassettes (molecular weight cut‐off, MWCO 3500, Pierce, USA). Subsequently, the solution was centrifuged two times at 18 000 rpm for 20 min. The final silk concentration was determined to be about 6–7 wt% by measuring the volume of the solution and the final dried weight.

##### Fabrication of CSFH

HRP, type VI (Sigma‐Aldrich, St. Louis, MO) lyophilized powder was dissolved in deionized water to make a stock solution at a concentration of 300 U mL^−1^. The HRP solution was added to the silk solution to a concentration of 10 U mL^−1^. To initiate gelation, 0.4 µL hydrogen peroxide (30%, Sigma Aldrich, St. Louis, MO) solution was added per mL of silk solution. Then the precursor can be functionalized with CNTs (single walled, length < 10 um) solution at a volume ratio of 1:4 (CNTs:precursor). Finally, the solution was mixed gently, and casted into a petri dish or a pre‐prepared mold at room temperature for ≈2 h.

##### SEM Image

The CSFH samples were quickly frozen using liquid nitrogen and then lyophilized until complete sublimation of water. All the micrographs were taken with a Hitachi S4800‐SEM.

##### Fabrication of Microneedle Arrays

A reverse silicon mold with inverted pyramid structure was first fabricated by photolithography and wet etching.1 mL concentrated silk solution (concentration: ≈15 wt%) was dropped onto the silicon mold with area of 2 cm × 2 cm and put into the vacuum chamber to remove bubbles. Subsequently the mold with solution was dried in fume hood for 12 h and then the microneedle array film can be peeled off.

##### Characterization of the CSFH‐Based Mechanical Sensor

The CSFH samples for tension test were pre‐immersed into DI water for 10 min. Strain and pressure measurements were acquired by a CMT4204 mechanical tester (SUST, China), while the resistances of the sensors were measured with a 34410A Agilent Digital Multimeter. Mechanical tests were conducted at a speed of 5 mm min^−1^. The CSFH samples for *I*–*V* curves measurement were pre‐immersed into DI water for 10 min for resistance uniformity. *I*–*V* curves were obtained by B2900 precision source/measure unit. To measure the resistance response to bending angles, the CSFH‐based sensor was installed on the index finger joint and different angles were formed by the finger. To measure the cycling stability of the CSFH pressure sensor, a liner motor was applied as the external force with a frequency of 5 Hz for >1400 s. Measurements were performed in a controlled temperature and humidity atmosphere (22 ± 1 °C and 50 ± 10% relative humidity).

##### Degradation in Different pH and Temperatures

For degradation, the CSFH precursor was doped with papain at a concentrate of 0.25% and set in the refrigerator of −4 °C for 12 h to generate the degradable CSFH. Nine CSFHs were cut into the same size (1 cm × 1 cm × 3 mm) and immersed into PBS solutions with pH = 6–8, respectively. The temperature gradients were supplied by water bath. After 1 h degradation, the CSFHs were taken out from PBS solutions and rinsed with DI water.

##### Laser Heating Triggered Degradation

For laser heating triggered degradation, the CSFH precursor was doped with papain at a concentrate of 0.25% and gold nanoparticles at a diameter of 20 nm. The CSFH network was peeled from a reverse mold printed by 3D printer. The CSFH network was then illuminated simultaneously by two sets of green lasers with incident radiation powers of 100 and 50 mW for 1 h. This measurement was performed in a controlled high humidity (90%).

##### On‐Skin Experiments

All human subjects involved in this context were fully volunteered and give written, informed consent before participation in the study. The CSFH‐based sensors with copper conductive adhesives as electrodes were attached onto the finger joints, knee joint, and neck, respectively by tightly wrapping adhesive tape to avoid any slipping of the mounted sensor.

##### ICP Measurement

All animal tests are approved by the Institutional Animal Care and Use Committee of Fudan University. For ICP measurements, Sprague‐Dawley rats (6–8 weeks old) were first anesthetized with an intraperitoneal injection of a ketamine/xylazine mix. After the craniotomy, the CSFH‐based mechanical pressure sensor was attached to the cortex surface and the cavity was sealed by bone cement for real‐time ICP monitoring. Then the surgical site was closed by the conventional suture in a standard process. In order to induce epilepsy in rats, anesthetized rats were injected with 2 mL PNG solution (1.6 × 10^5^ U mL^−1^). After 30 min monitoring, the rats were conducted euthanasia.

##### In Situ Controlled Therapy of Epilepsy

BALB/c Nude mice (6 weeks old) were first anesthetized with an intraperitoneal injection of a ketamine/xylazine mix. Then the controlled degradable CSFH‐based mechanical sensor, doped with phenobarbital (2 mg mL^−1^), was adhered tightly to silk microneedle film using concentrated silk solution as glue. The combined CSFH patch was installed on the back of the mouse to monitor its breathing and convulsion during the epilepsy.

In order to induce epilepsy, anesthetized mice were injected with 200 µL PNG solution (1.6 × 10^5^ U mL^−1^). Once the epilepsy symptom was monitored by the CSFH‐based sensor, a 532 nm laser heating was used to illuminate the CSFH to trigger its degradation and consequent the therapeutic drug release. This measurement was performed in a controlled high humidity (90%).

## Conflict of Interest

The authors declare no conflict of interest.

## Supporting information

Supporting Information
