# Supplementary material for: Body‐Integrated, Enzyme‐Triggered Degradable, Silk‐Based Mechanical Sensors for Customized Health/Fitness Monitoring and In Situ Treatment
Source: Adv Sci (Weinh). 2020 May 11;7(13):1903802. doi: 10.1002/advs.201903802 (PMC7341100; doi:10.1002/advs.201903802)
Supplement: Supplementary file 1 — Supporting Information [file ADVS-7-1903802-s001.pdf]

Copyright WILEY-VCH Verlag GmbH & Co. KGaA, 69469 Weinheim, Germany, 2020.

Supporting Information

Body-integrated, Enzyme-triggered Degradable, Silk-based Mechanical Sensors for Customized Health/Fitness Monitoring and In Situ Treatment

*Shan Zhang†, Zhitao Zhou†, Junjie Zhong†, Zhifeng Shi, Ying Mao, and Tiger H. Tao**

Shan Zhang, Dr. Zhitao Zhou, Prof. Tiger H. Tao
State Key Laboratory of Transducer Technology, Shanghai Institute of Microsystem and Information Technology, Chinese Academy of Sciences, Shanghai 200050, China.
E-mail: tiger@mail.sim.ac.cn

Prof. Tiger H. Tao
Center of Materials Science and Optoelectronics Engineering, University of Chinese Academy of Sciences, Beijing 100049, China

Shan Zhang, Prof. Tiger H. Tao
School of Graduate Study, University of Chinese Academy of Sciences, Beijing 100049, China

Dr. Junjie Zhong, Dr. Zhifeng. Shi, Prof. Ying Mao
Department of Neurosurgery, Huashan Hospital of Fudan University, Shanghai, 200040, China

Prof. Tiger H. Tao
School of Physical Science and Technology, ShanghaiTech University, Shanghai 200031, China

Prof. Tiger H. Tao
Institute of Brain-Intelligence Technology, Zhangjiang Laboratory, Shanghai 200031, China

Prof. Tiger H. Tao
Shanghai Research Center for Brain Science and Brain-Inspired Intelligence, Shanghai 200031, China

Keywords: Silk Hydrogels, Degradable, Mechanical Sensor, Health Monitoring, In Situ Treatment

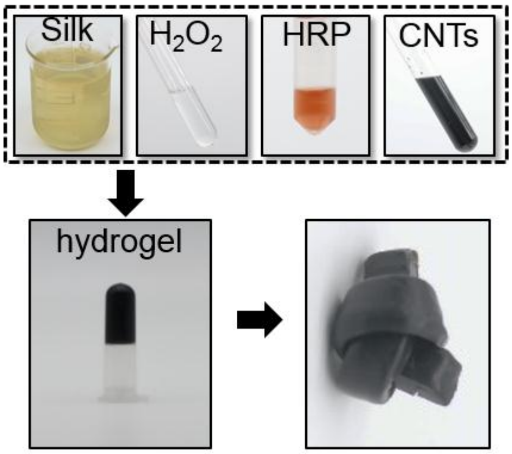

Figure S1. Fabrication of the CSFH. Silk, hydrogen peroxide, horseradish peroxidase (to generate elastic hydrogels), and CNTs solutions were mixed and casted in a petri dish for ~2 h.

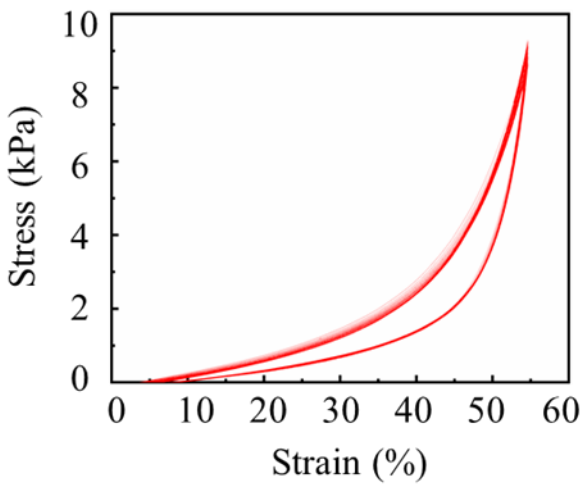

Figure S2. The stress-strain curves of compression tests for 100 cycles. 100 curves almost overlap.

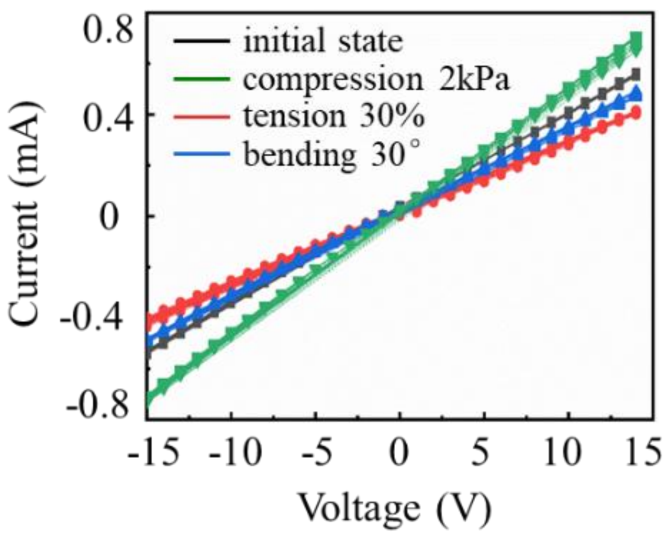

Figure S3. 100 cycles of current–voltage curves of the CSFH under different deformations.

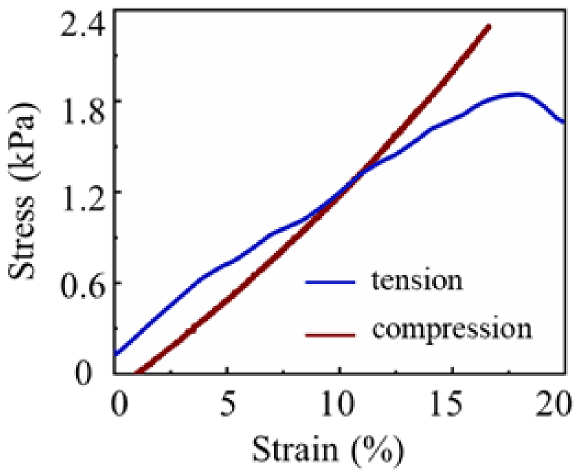

Figure S4. The stress-strain curves of the CSFH samples upon compression and tension without being pre-immersed into DI water. The tension modulus is close to the compression modulus in the low strain region.

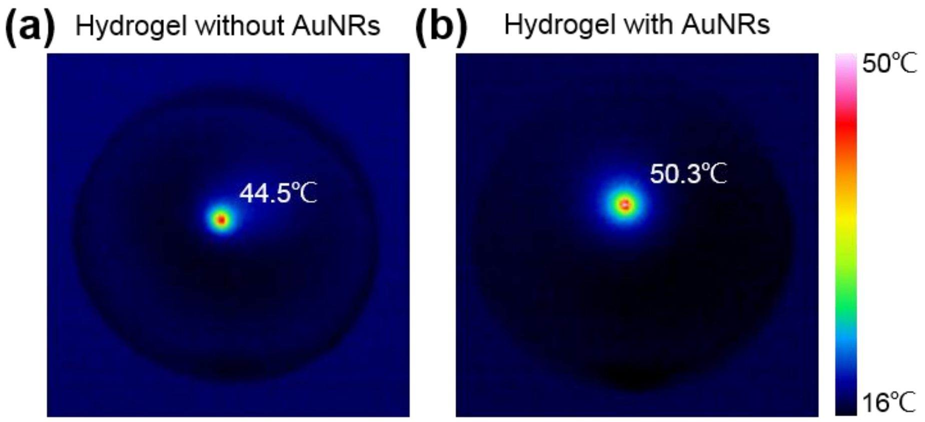

Figure S5. Laser heating-induced temperature increase in the CSFH with AuNRs and without AuNRs: The CSFHs were illuminated for 5 min using laser beam of 532 nm with a radiation power of 100 mW to reach a stable equilibrium temperature. The temperature reaches (a) 44.5 ºC for CSFH without AuNRs and (b) 50.3 ºC for CSFH with AuNRs.

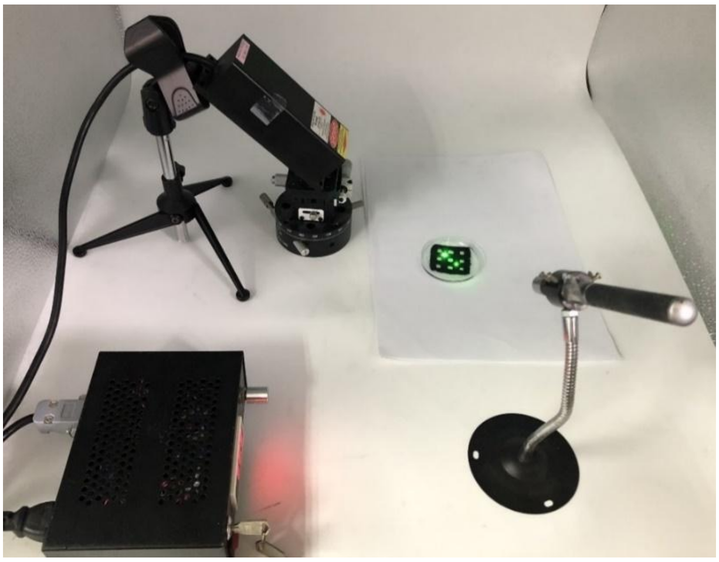

Figure S6. Experimental setup of hydrogel degradation controlled by laser with different radiation power.

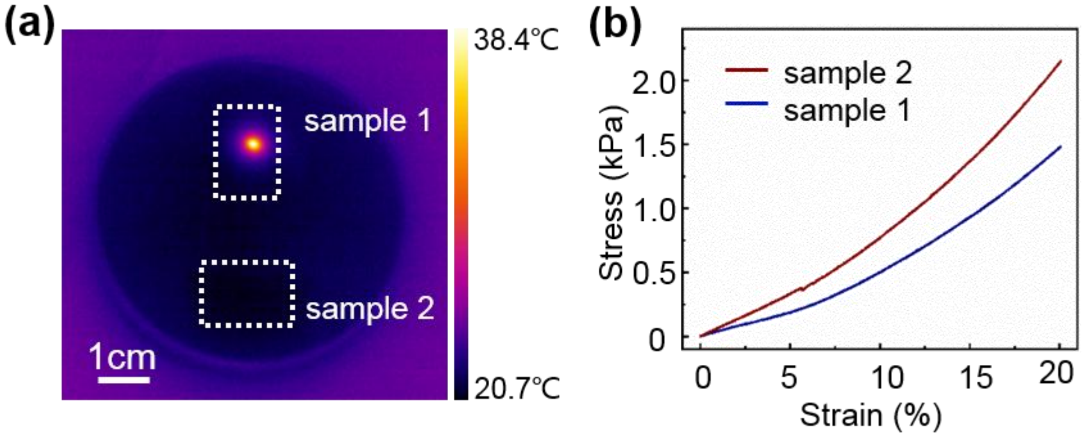

Figure S7. Mechanical property contrast between the CSFH samples with and without laser illumination. (a) The thermal image of two samples illuminated for 10 min and 0 min, respectively. (b) Stress-strain curves of two samples.

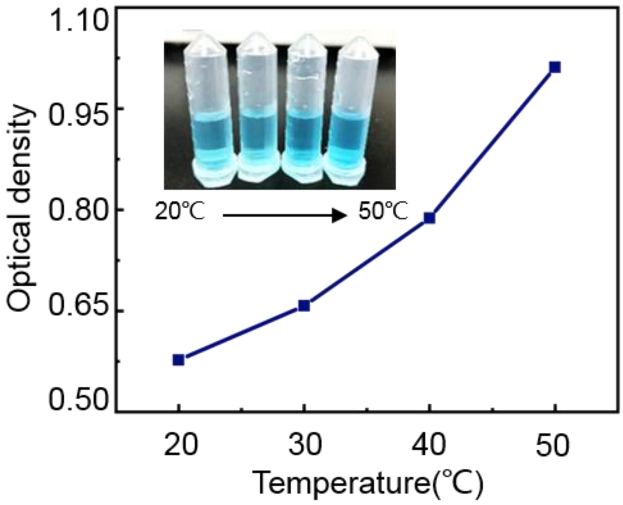

Figure S8. Simulative measurement of drug (using blue pigment) release rates at different temperatures. Pigment-doped CSFHs were degraded at different temperatures. The drug release rates at different temperatures were analyzed by measuring the optical density (corresponding with concentration) of solutions after degradation.

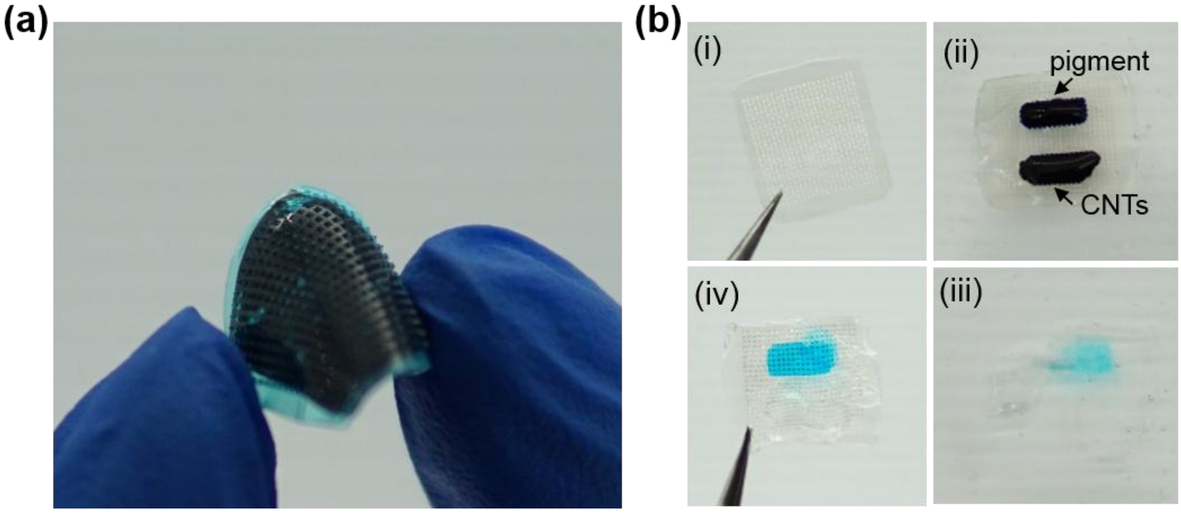

Figure S9. Simulative experiment of silk microneedles film serving as a filter to CNTs (length: ~10um) and phenobarbital ($C_{12}H_{12}N_2O_3$, molecular weight: 232, <10nm). The blue pigment ($C_{37}H_{34}N_2Na_2O_9S_3$, molecular weight: 792.85, <10nm) was used to simulate Phenobarbital because pigment is visible. (a) Photograph of the patch loaded with a blue pigment. (b) i) A clean silk microneedle film placing on the PDMS substrate. ii) Dropping 10 ul pigment solution and 10 ul CNTs solution on the film. iii) The photograph of PDMS substrate when the microneedle film was removed after 30 min. The result shows that only blue pigment can penetrate the microneedle film onto the PDMS substrate, because that CNT is large molecule so it could not penetrate the microneedle film while blue pigment is small molecule that could go through the microneedles film. iv) Only residual blue pigment remains on the microneedle film after inserted into DI water for 10 min.
